# Supplementary material for: Time‑resolved multi-omic analysis of paclitaxel exposure in human iPSC‑derived sensory neurons unveils mechanisms of chemotherapy‑induced peripheral neuropathy
Source: Cell Death Dis. 2026 Feb 10;17(1):211. doi: 10.1038/s41419-026-08445-2 (PMC12921266; doi:10.1038/s41419-026-08445-2)
Supplement: Supplementary file 15 — Dataset 1. [file 41419_2026_8445_MOESM15_ESM.zip › CDD_Open_Data/04_RNA_Seq/Timeline_panel_plots_etc_2023-01-23_1609.zip!/Panel_Plots_etc/Evidence_Plots/post5d/evidence_plot_standard contrasts_msigdb_reactome_5d_PTX_vs_DMSO_ID6_M27616_REACTOME_NEUREXINS_AND_NEUROLIGINS.pdf]

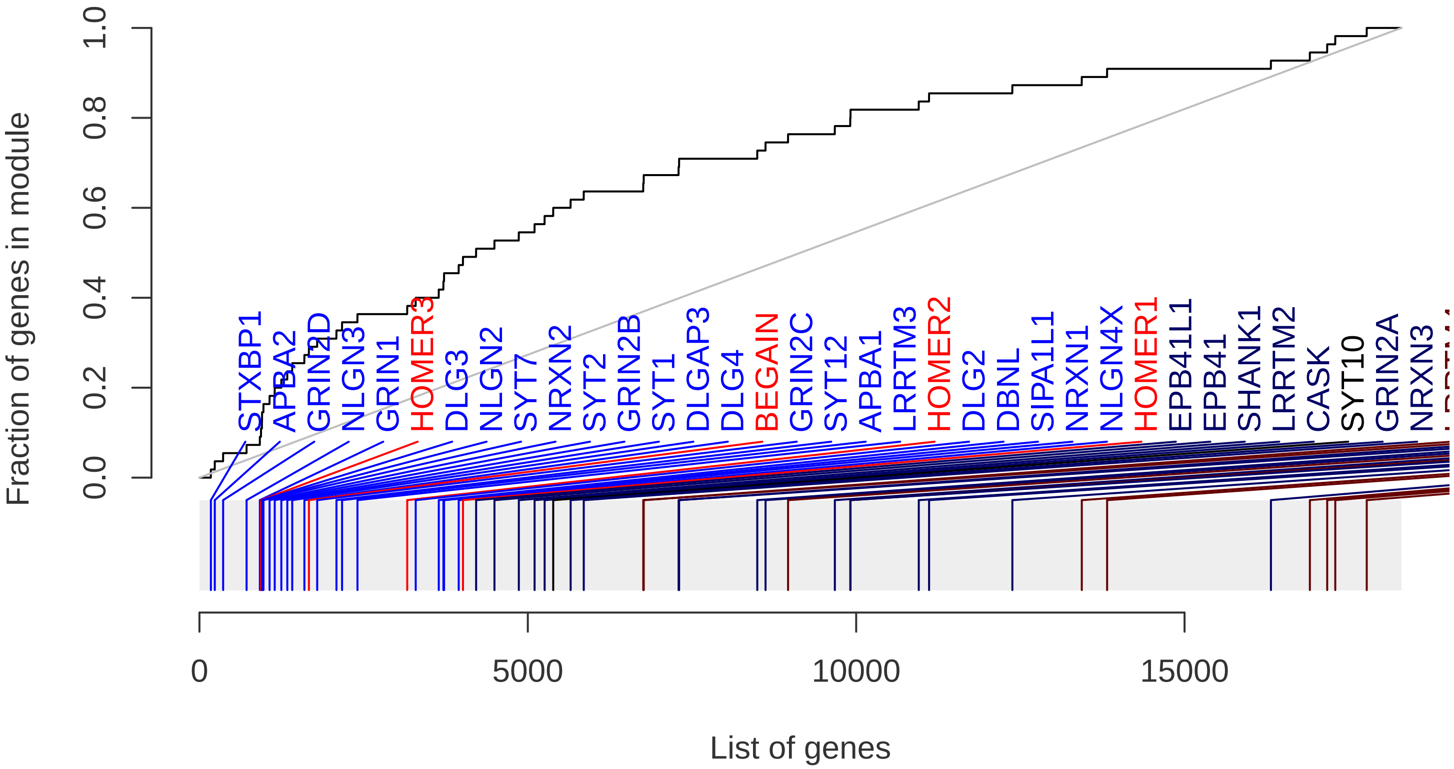

Fraction of genes in module
0.0
0.2
0.4
0.6
0.8
1.0
STXBP1
APBA2
GRIN2D
NLGN3
GRIN1
HOMER3
DLG3
NLGN2
SYT7
NRXN2
SYT2
GRIN2B
SYT1
DLGAP3
DLG4
BEGAIN
GRIN2C
SYT12
APBA1
LRRTM3
HOMER2
DLG2
DBNL
SIPA1L1
NRXN1
NLGN4X
HOMER1
EPB41L1
EPB41
SHANK1
LRRTM2
CASK
SYT10
GRIN2A
NRXN3
0
5000
10000
15000
List of genes
